# Improved Chou-Fasman method for protein secondary structure prediction

**DOI:** 10.1186/1471-2105-7-S4-S14

**Published:** 2006-12-12

**Authors:** Hang Chen, Fei Gu, Zhengge Huang

**Affiliations:** 1 Department of Biomedical Engineering, College of Biomedical Engineering and Instrument Science, Zhejiang University, Hangzhou, 310027, China; 2 Department of Biotechnology, College of Life Sciences, Zhejiang University, Hangzhou, 310027, China; 3 Department of Computer Science, Center for engineering and scientific computation, Zhejiang University, Hangzhou, 310027, China

## Abstract

**Background:**

Protein secondary structure prediction is a fundamental and important component in the analytical study of protein structure and functions. The prediction technique has been developed for several decades. The Chou-Fasman algorithm, one of the earliest methods, has been successfully applied to the prediction. However, this method has its limitations due to low accuracy, unreliable parameters, and over prediction. Thanks to the recent development in protein folding type-specific structure propensities and wavelet transformation, the shortcomings in Chou-Fasman method are able to be overcome.

**Results:**

We improved Chou-Fasman method in three aspects. (a) Replace the nucleation regions with extreme values of coefficients calculated by the continuous wavelet transform. (b) Substitute the original secondary structure conformational parameters with folding type-specific secondary structure propensities. (c) Modify Chou-Fasman rules. The CB396 data set was tested by using improved Chou-Fasman method and three indices: Q3, Qpre, SOV were used to measure this method. We compared the indices with those obtained from the original Chou-Fasman method and other four popular methods. The results showed that our improved Chou-Fasman method performs better than the original one in all indices, about 10–18% improvement. It is also comparable to other currently popular methods considering all the indices.

**Conclusion:**

Our method has greatly improved Chou-Fasman method. It is able to predict protein secondary structure as good as current popular methods. By locating nucleation regions with refined wavelet transform technology and by calculating propensity factors with larger size data set, it is likely to get a better result.

## Background

Protein sequence determines its senior structures [1]. Based on this hypothesis, the protein secondary and tertiary structures and their domains are contained within a peptide chain. The protein secondary structure has been studied intensely, since it is very helpful to reveal the functions of protein with unknown structures. In addition, it has been shown that the prediction of protein secondary structure is a step toward protein 3-dimensional structure prediction and it can also be included in threading method to identify distantly related proteins [2].

Many efforts have been made to extract useful information of protein secondary structure from sequences [3-10]. Among them, Chou-Fasman method (CFM) [10] is one of the pioneer works and it is still widely used. It is convenient to use with many merits. It is an empirically statistical method by assigning a set of prediction values to a residue and by applying a simple algorithm. Three rules have been proposed in CFM, including the locating of nucleation regions, extending nucleation regions, and the refinement of secondary structure segment [10].

With further investigations on CFM, it has been found that there were three critical deficiencies in CFM. First, the Chou-Fasman parameters are unreliable [11,12]. Since CFM is a statistics-based method, it is very important to perform statistics with a large number of data set in order to get reasonable confidence. However, in their work, Chou and Fasman only calculated 15, 29, 64 proteins in 1974 [10], 1978 [3] and 1989 [13], respectively. The limited size of data set might due to the small number of non-homologous proteins with solved three-dimensional structures at that time. However, as a result, it causes the wide confidence limit which even makes us difficult to tell if an amino acid is a helix former or breaker [11]. Secondly, the accuracy of CFM is low. There are several different versions on how accurate the CFM is. Chou and Fasman quoted the accuracy over 70% using their method. However, most researchers considered the number is 50–60% [10,14,15]. The difference which makes people doubt the consequence from Chou and Fasman derives from the test data set. Chou and Fasman used their training data as the test data, while other researchers used different types of test data [10]. It implies that cross-validation technique, where test proteins are removed from the training set, is a more realistic evaluation of protein secondary structure prediction accuracy to be obtained [16]. Thirdly, CFM trends to over predict in helix and strand and under predict in coil. It indicates that many coil positions are incorrectly predicted as helices or strands in CFM that causes high false positive in CFM.

In order to solve the problems mentioned above, people (include Chou and Fasman themselves) have produced a lot of modifications in the past few decades. Their works are focused on the modification of amino acid conformation propensities [11-13,17-20] since this is the key point to improve CFM (as we all know that the location of nucleation and the threshold of extension are closely related to the residue conformation propensities). In addition, the propensity factors were used in several different protein secondary structure prediction methods [21,22]. Among these different kinds of propensities calculated by various methods, most of them examined amino acid secondary structure propensities in a whole conformational base regardless of protein folding types. However, it has been found that proteins of 4 major folding classes (all α-Helical, all β-Sheet, α/β, and α+β, classified by Levitt and Chothia [23]) are different in folding, packing and so on [24]. Moreover, it has been proven that folding class of certain protein is related to its amino acid [25], and the knowledge of protein folding class is useful in improving accuracy of protein secondary structure prediction [26]. These researches demonstrate that the amino acids' secondary structure parameters are different among the four folding types. Fortunately, Jiang et al. have calculated the propensities by calculating the proteins with different classes [20]. The similarity among all these sequences is less than 30%, and they have proven that their results are statistically significantly different with confidence level of 90%. That is, their data set is both non-homologous and large enough. That is, these parameters are reliable.

Besides statistic methods, there are several sequence analysis approaches proposed for protein secondary structure prediction based on the physicochemical property of residues. Wavelet transform (WT) technology based on hydrophobicity values is one of them. WT is a local time-frequency analysis method with both time window and frequency window changeable. Because of its character of multi-resolution, WT has been applied in bioinformatics to analyze and process biological data [27] recently. To deal with protein sequence, WT coefficients with different scale parameters correspond to different structural hierarchies [28]. Being the numerical basis of WT, hydrophobicity value plays an important role in the method. Hydrophobicity, one of the protein significant properties, makes the water-fearing side chain to crimp into a compressed conformation to avoid the water phase [29]. This configuration is important for the existence and stability of protein 3-dimensional structures. Hence, we can predict protein secondary structure on a hydrophobicity basis.

Many hydrophobicity values have been provided [30,31]. Based on one of them, Mandell et al. [28] have found that the number of protein secondary structure segments is related to the coefficients cycles at certain scale calculated by continuous wavelet transform (CWT). This method is good at determining the number of secondary structure segments and locating the regions of them, which is a weak point of CFM. However, the accurate prediction of the secondary structure conformation of every single residue is a problem for CWT.

By comparing the advantage and disadvantage between CFM and hydrophobicity based CWT method, it can be inferred that they are almost complementary for each other. The nucleation regions calculated by CWT seems to be better than CFM, while the Chou-Fasman extension rule is a good solution for the fine prediction of CWT.

In this paper, we improved the CFM with the technology mentioned above. In addition, we found the merit and shortage of this renewed CFM by comparing it with some current popular methods. Finally, we realized the full-automation of our method for the analysis of great number of data set.

## Methods

Chou-Fasman rules can be concluded in three points below [10]:

1. Forming of nucleation. A nucleation can be predicted when 4 of 6 sequential residues in certain segment tend to form helix (the helix former), and this number is 3 of 5 for strand.

2. The nucleation regions are extended along both directions of the sequence until the average 4-peptides propensities drops below 1.

3. If any extended segment with average propensities ⟨*P*_α_⟩ > 1.03(helical propensities larger than 1.03 are strong alpha former and alpha former [3]) and ⟨*P*_α_⟩ > ⟨*P*_β_⟩ (subscript α means helical propensities while β corresponds to strand propensities), it can be predicted as helix. And the condition changes to ⟨*P*_β_⟩ > 1.05 (strand propensities larger than 1.05 are strong strand former and strand former [3]) and ⟨*P*_β_⟩ > ⟨*P*_α_⟩ for strand. If both helix and strand are predicted in certain region (overlapped region), the secondary structure conformation with higher average propensities is predicted.

In our research, all three rules were improved with three steps. First, the hydrophobicity value based CWT technology was used to calculate the number and locations of protein secondary structure segments, and then substituted the nucleation regions of CFM with these positions. To improve the second rule, folding type-specific structure propensities were used instead of traditional Chou-Fasman parameters to extend the secondary structure segments and to deal with the overlapped regions. We just undid some processes in modification of the third rule.

### CWT for nucleation regions

To explain this improvement, a sample protein was selected randomly from Protein Data Bank (PDB) with ID 3dfr. From its data file, the sequence information and secondary structure information were extracted. The referenced secondary structure for each position was defined by DSSP [32]. According to this dictionary, we classified secondary structure information into 3 classes: H, G, and I are helices; E and B are strands; other conformations are coils.

**Figure 1:**
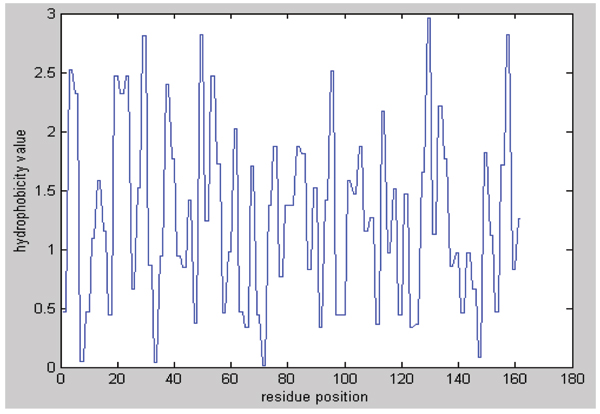
Hydrophobicity sequence of protein 3dfr.

Then the character sequence must be converted into its corresponding hydrophobic values (Figure 1). The values obtained by Mandell et al. [28] were adopted, listed in Table 1.

**Figure 2:**
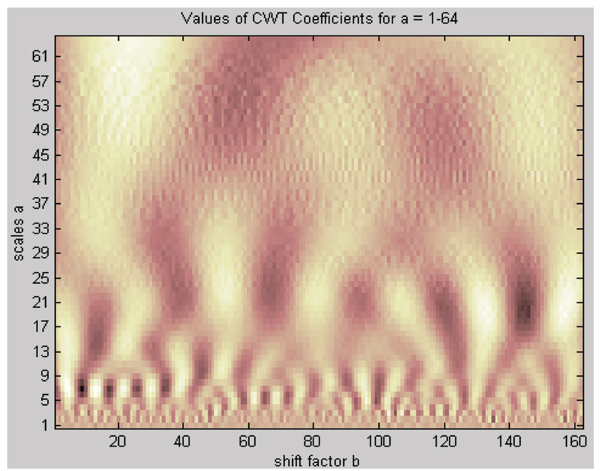
Plot of Morlet wavelet transform of the amino acid hydrophobic free energy sequence at scales from 1 to 64 (dark represents coefficient at minimum value whereas light correspond to maximum value).

To analyze the numerical sequence in different scales, 1-D continuous wavelet transform (CWT) was used with scale ranging from 1 to 64 (Figure 2). Here we chose the Morlet function (equation 1) as mother wavelet due to its symmetry, finity and continuity.

Ψ(*x*) = *C *exp(-*x*^2^/2)cos5*x *    (1)

where C is a constant, and in our method we chose 1 for convenient calculation.

The continuous wavelet transform for a function f(t) is defined as:



where *a *is the scale parameter, *b *is the translation parameter (*a *> 0, *b *∈ R).

The hydrophobic cycle, as defined in reference 28, consists of one dark band and one light band (In Figure 2, dark dots represented for the coefficients at minimum value whereas light ones corresponded to maximum value. That means the light bands are more hydrophobic than the dark ones. To determine dark or light with numerical values, the coefficients at certain scale should be picked up. The position with coefficient > 0 was considered as the light part whereas dark part represented for the coefficient < 0). Examining Figure 2 in the scale dilation axis at the region of approximate 9, we could count 15 hydrophobic cycles along the length of the protein. It means there are 15 secondary structure elements (α-helix and β-strand) in 3dfr. Comparing with the secondary structure information derived from DSSP (17 secondary structure units), we found that the number was very close.

**Figure 3:**
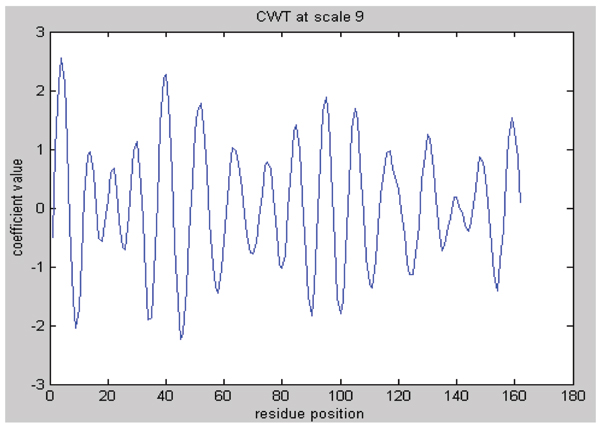
Plot of CWT coefficients at scale 9.

We judged this number as the same value as that of nucleation region. Moreover, we supposed that every cycle contained a nucleation region. In this article, positions with local extreme value (including maximum and minimum values for the reason explained in the discussion part) at scale 9 of CWT were considered as nucleation sites. Wavelet coefficients at scale 9 were shown in Figure 3.

We must improve the CWT formula since the sequence chain is discrete. The formula was altered as follows (the sign "≈" means approximately equal to):

Suppose *f*_1_(*t*) = *s *[*k*], *t *∈ [*k*, *k *+ 1), then



Hence, the coefficients of CWT can be calculated by the difference of convolution of s [k] and the integral formula .

According to the analysis above, *a *was set as 9. And we took the positions with local extreme value as the nucleation sites.

### Extend with folding type-specific structure propensities

The folding type-specific conformation propensities had been divided into 4 groups (corresponding to the 4 protein classes): 59 proteins in α class, 76 proteins in β class, 40 proteins in α+β class, and 52 proteins in α/β class, respectively. All these data had a sequence similarity less than 30%, and the data set size was large enough to get the confidence level of 90% [20]. Hence, these parameters are reliable enough to be used in protein secondary structure prediction. The four class propensities are shown in Table 1 of reference 20.

The extension rule is related not only with propensities, but also with the terminating threshold. This value is 1 for both helix and strand in CFM, which is approximately the average propensity value of the 20 amino acids. However, with the use of folding type-specific propensities in our method, this threshold should be modified for both helix and strand. We tested sequential 5 numbers beside the average number with the interval of 0.01. The best one with high accuracy of all the evaluation indices was adopted (shown in the results part in detail).

### Refinement

We only reserved the process of overlapped secondary structure segments and abandoned others since those empirical rules are not suitable for the data set now. We also didn't bring in the helix/strand breakers which were used to abolish secondary structure segment in Chou-Fasman rules. This is because the breaker such as proline was found to be existed in helix or strand of some proteins.

### Data set and evaluation indices

The data set CB396 (please see Supplementary file "dataset.pdf"), proposed by Cuff and Barton [33], was used to test our algorithm for two reasons: (a) It is a non-redundant sequence set derived from a sensitive sequence comparison algorithm and cluster analysis combining with filtering the X-ray crystal structures with resolutions over 2.5 Angstroms; (b) This test data set was totally different from the training set which was used in the calculation of folding type-specific structure propensities. Hence, it is suitable for cross validation of our method with more realistic evaluation of prediction accuracy to be obtained [34]. We classified this data set into four classes based on the protein structural classification database SCOP [35].

Three commonly used indices were adopted to assess our method. Two traditional indices, Q_3 _and Q_pre _were used to evaluate the accuracy of individual residues and the degree of over predict, respectively [36]. Another index which was proposed recently is the SOV (segment overlap measure). It was used to measure the accuracy of secondary structure segments [37]. And in our method, the SOV index was concerned a lot since it is more realistic and significant in measuring protein secondary structure prediction method.

In order to specify the efficiency of our algorithm, the indices derived from our method were compared with four currently popular methods. All these methods are based on different technologies. The DSC [6] algorithm is based on GOR [4] and multiple sequence alignment, NNSSP [7] is a scored nearest-neighbor method, PHD [8] is based on artificial neural network, and PREDATOR [9] uses local sequence alignment approach.

All the observed secondary structures (derived from PDB crystal structure and DSSP protein secondary structure dictionary) and predicted secondary structures (calculated by our method and four other methods) were performed with two processes: (a) Helix segment with residue number less than 3 is removed and considered as coil since it is unable to form helix with residues number < 3. (b) Strand segment with residue number less than 2 is considered as coil. The refined results were biologically significant. With this process, the accuracy of the four current methods in our calculation is a little different from the results computed in reference 33.

**Figure 4:**
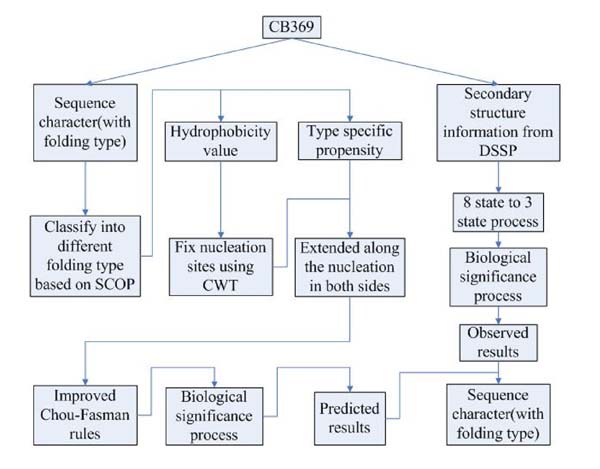
Algorithm flowchart with prediction and evaluation.

The whole algorithm flowchart is shown in Figure 4, with both prediction part and evaluation part. People who are interested in this algorithm can contact us by sending an email requesting source code (written in matlab language).

## Results

Before performing our method, we compared traditional CFM (proposed in 1978) with four current methods mentioned above to see how large the difference is. The result is shown in Table 2. It can be found that the difference between them is tremendous. Nearly all the indices in CFM are less than other four methods, and most of them are 20–30 percent lower, especially for the SOV and Q_pre _indices. That means the CFM is weak in hitting the protein secondary structure segment and it tends to over predict.

Every step of our method mentioned above was tested to see if these modifications are efficient.

First the nucleation regions were calculated with continuous wavelet transform (CWT) rather than performing Chou-Fasman rule 1. The result is shown in Table 3. From these values, it can be found that many indices were no big difference but the SOV indices were improved increasingly.

By performing the second step of our method with setting the extension threshold to 1 as used in CFM, it can be found from Table 4 that all the indices (except Q_E_) have been increased distinctly. This result again proved that the propensities are very important for CFM.

With the modification of Chou-Fasman third rule, we found that the SOV indices were improved while the Q_pre _indices were a little worse than CFM. We reserved this modification because the SOV indices were considered more important in our method. The result was shown in Table 5.

The degree of improvement with 3 different steps of our method was shown in Table 6. From this result, it can be found that each modification has improved several indices while other indices are nearly invariant. Hence, we are confident to believe that with the combination of all three modifications, the accuracy should be much better than CFM for all the indices. Furthermore, we have to change the extension threshold since the Chou-Fasman parameters had been substituted with folding type-specific propensities. In our method, we calculated the 5 threshold beside the average propensity value for proteins of the four classes, with interval of 0.01. By considering the overall indices especially the SOV, we got the best values which were listed in Table 7. However, the results calculated by different thresholds around average propensity value were very close in our test.

The final result was shown in Table 8. And it could be found that our method has a great improvement in every index, about 15–20% better than CFM in accuracy. Comparing our method with four current methods, some indices in our method were better, while some were close or worse as shown in Table 9. However, in general, our method is comparable with these four current popular methods.

## Discussion

By use of cross validation, all the results calculated in this article are reliable. We utilized some parameters concluded by other researchers [10,20,28], and ensured that our test data set is different from their training data set. There is one exception, the extension threshold. The same data set was used to train and test this value. However, as we mentioned in the results part, this number is around the average propensity value with no significant difference in the final result. Hence, it is unnecessary to calculate the extension threshold using cross validation, any number around the average propensity value is accepted.

In our method, we took the positions with local extreme value of every cycle of coefficients at certain scale calculated by CWT as the nucleation regions. This is for 2 reasons: (a) The extreme value corresponds to the singularity point in CWT which is considered as one of the most important parts in analysis of CWT. (b) The residue hydrophobicity in secondary structure segments is alternate. When helix or strand is buried inside of a protein, their residues are more hydrophobic. If the secondary structure component is located on the surface of a protein, their residues are usually hydrophilic.

The advantage of our method can be concluded in 3 points below:

1. Our method has inherited the merit of CFM. It is very simple and easy to realize. It is also fast and low computational consumption although the CWT method had been brought in our method because it doesn't need to do training and sequence alignment.

2. Our method has solved two problems in CFM, the unreliable parameters and low accuracy. And the problem over prediction has been partially solved.

3. Our method has a great improvement in all of the indices compared with CFM, and the result of our method is comparable with current popular methods.

However, there are still several problem existed in our method:

1. The high false positive still existed in our method. By investigating the indices with esign '-' (which means our method has low accuracy at these indices) from Table 9, we can conclude that our method was trended to over predict in helix and strand while under predict in coil, this leads to high accuracy in helix or strand indices and low accuracy in overall indices (Q_3_, SOV). Another conclusion is that the hit rate of secondary structure segment (nucleation) in our method was not high enough, and this blocks the increase of SOV indices.

2. In our method, protein class must be obtained first since the propensities are assigned according to protein class. This transcendental condition has narrowed the application area of our method. However, by use of sequence alignment, the class of protein with unknown structure may be decided.

3. In folding type-specific structure propensities, there is no strand value in proteins with all alpha class, while no helix value in all beta class. However, in SCOP database, protein in all alpha class may still contain strand segments, while in all beta class, helix segments can be found. Hence, the strand propensity in alpha class and helix propensity in beta class need to be calculated. Nevertheless, in our method, the accuracy of alpha class and beta class is still well. This may be due to the small proportion of strand in alpha class and low proportion of helix in strand class.

To deal with these problems, further modifications are needed to improve our method:

1. Nucleation regions must be refined since they are very important in CFM. If we can hit every protein secondary structure segment nucleation, the result should be improved increasingly. It may be a possible way to solve this problem by using CWT to look for the scale of helix, strand, and coil, respectively.

2. Improve the calculation method of propensity. In our method, we used the propensities which were computed based on statistics. However, for more biological significance, it is helpful to calculate propensities by use of physiochemical technology. For example, the thermodynamic method which was used in reference [17] and [18]. In addition, coil propensities can be included in protein secondary structure prediction for reducing over prediction.

3. To strict the extension threshold. This modification may need a large number of statistics.

4. Develop new technique and rule to treat with breakers. This is an efficient way to solve over prediction.

## Conclusion

In our method, CFM was improved with modifications in nucleation regions, parameters and some rules. One represented data set and 3 different kinds of indices were used to evaluate our method. The results have showed that our method has greatly improved CFM. It is also comparable with current popular methods in protein secondary structure prediction. With the further improvement mentioned above, it is reasonable to believe that our method is able to predict protein secondary structure with high accuracy.

## Figures and Tables

**Table 1:** The hydrophobic values of 20 amino acids

amino acid	value	amino acid	value
Gly	0.00	Cys	1.52
Gln	0.00	Lys	1.64
Ser	0.07	Met	1.67
Thr	0.07	Val	1.87
Asn	0.09	Leu	2.17
Asp	0.66	Tyr	2.76
Glu	0.67	Pro	2.77
Arg	0.85	Phe	2.87
Ala	0.87	Ile	3.15
His	0.87	Trp	3.77

**Table 2:** Compare traditional CFM with four current methods

Method	Q_3_(%)	Q_H_(%)	Q_E_(%)	Q_H_^PRE ^(%)	Q_E_^PRE ^(%)	SOV (%)	SOV_H _(%)	SOV_E _(%)
CFM	46.88	55.64	60.77	49.22	34.40	36.26	42.63	43.95
DSC	69.10	63.65	54.95	73.70	71.31	66.19	63.14	60.86
NNSSP	72.31	64.93	55.28	80.42	73.34	67.32	66.07	63.40
PHD	72.60	65.38	68.59	77.98	63.55	69.94	65.74	72.05
PREDATOR	69.58	62.21	54.50	75.33	70.14	64.85	64.23	60.57

**Table 3:** Result with the improvement of nucleation

Method	Q_3_(%)	Q_H _(%)	Q_E_(%)	Q_H_^PRE ^(%)	Q_E_^PRE ^(%)	SOV (%)	SOV_H _(%)	SOV_E _(%)
CFM	46.88	55.64	60.77	49.22	34.40	36.26	42.63	43.95
Improved nucleation	48.10	52.14	58.30	49.54	34.71	40.82	44.97	47.82

**Table 4:** Result with the improvement of propensities

Method	Q_3_(%)	Q_H _(%)	Q_E _(%)	Q_H_^PRE ^(%)	Q_E_^PRE ^(%)	SOV (%)	SOV_H _(%)	SOV_E _(%)
CFM	46.88	55.64	60.77	49.22	34.40	36.26	42.63	43.95
Improved propensity	54.56	57.14	60.14	69.70	55.48	40.41	43.93	46.52

**Table 5:** Result with the improvement of Chou-Fasman rules

Method	Q_3_(%)	Q_H_(%)	Q_E_(%)	Q_H_^PRE ^(%)	Q_E_^PRE ^(%)	SOV (%)	SOV_H _(%)	SOV_E _(%)
CFM	46.88	55.64	60.77	49.22	34.40	36.26	42.63	43.95
Improved rules	44.09	57.72	72.21	45.86	31.11	36.51	46.06	51.86

**Table 6:** The degree of improvement with 3 different steps of our method

	Q_3_	Q^PRE^	SOV
Step 1	No difference	No difference	Distinct better
Step 2	No difference	Much better	A little better
Step 3	No difference	A little worse	Distinct better

**Table 7:** Extension threshold for proteins of 4 classes

Protein Class	helix extension threshold	strand extension threshold
All alpha	0.98	No statistic
All beta	No statistic	1.01
Alpha and beta (α/β)	1	1.02
Alpha or beta (α+β)	0.99	0.98

Notice that the strand extension threshold in all alpha class and the helix extension threshold in all beta class are not statistical in reference 20.

**Table 8:** Result with all three improvements

Method	Q_3_(%)	Q_H_(%)	Q_E_(%)	Q_H_^PRE ^(%)	Q_E_^PRE ^(%)	SOV (%)	SOV_H _(%)	SOV_E _(%)
CFM	46.88	55.64	60.77	49.22	34.40	36.26	42.63	43.95
With all improvements	56.10	72.86	68.17	67.17	53.35	51.14	60.89	57.46

**Table 9:** Compare our method with 4 current methods

Method	Q_3_(%)	Q_H_(%)	Q_E_(%)	Q_H_^PRE ^(%)	Q_E_^PRE ^(%)	SOV (%)	SOV_H _(%)	SOV_E _(%)
Our method	56.10^-^	72.86*	68.17*	67.17^+^	53.35^-^	51.14^-^	60.89^+^	57.46^+^
DSC	69.10	63.65	54.95	73.70	71.31	66.19	63.14	60.86
NNSSP	72.31	64.93	55.28	80.42	73.34	67.32	66.07	63.40
PHD	72.60	65.38	68.59	77.98	63.55	69.94	65.74	72.05
PREDATOR	69.58	62.21	54.50	75.33	70.14	64.85	64.23	60.57

Superscript marker with sign '*', '+', '-' means the accuracy of our method was better than, close to, worse than other four methods in these indices, respectively.

## References

[B1] Anfinsen CB, Haber E, Sela M (1961). White F.H. The kinetics of the formation of native ribonuclease during oxidation of the reduced poly peptide chain. Proc Natl Acad Sci.

[B2] Rost B, Schneider R, Sander C (1997). Protein fold recognition by prediction-based threading. J Mol Biol.

[B3] Chou PY, Fasman GD (1978). Prediction of the secondary structure of proteins from their amino acid sequence. Adv Enzymol Relat Areas Mol Biol.

[B4] Garnier J, Osguthorpe DJ, Robson B (1978). Analysis and implications of simple methods for predicting the secondary structure of globular proteins. J Mol Biol.

[B5] Holley LH, Karplus M (1989). Protein secondary structure prediction with a neural network. Proc Natl Acad Sci.

[B6] King RD, Saqi M, Sayle R (1997). Sternberg M.J. DSC: Public domain protein secondary structure prediction. Comut Appl Biosci.

[B7] Salamov AA, Solovyev VV (1995). Prediction of protein secondary structure by combining nearest-neighbor algorithms and multiple sequence alignments. J Mol Biol.

[B8] Rost B (1996). PHD: Predicting one-dimensional protein structure by profile-based neural networks. Methods Enzymol.

[B9] Frishman D, Argos P (1997). Seventy-five percent accuracy in protein secondary structure prediction. Proteins.

[B10] Chou PY, Fasman GD (1974). Prediction of protein conformation. Biochemistry.

[B11] Kabsch W, Sander C (1983). How good are predictions of protein secondary structure?. FEBS Lett.

[B12] Kyngas J, Valjakka J (1998). Unreliability of the Chou-Fasman parameters in predicting protein secondary structure. Protein Engineering.

[B13] Chou PY, Fasman GD (1989). Prediction of Protein Structure and the Principles of Protein Conformation.

[B14] David MountW (2002). Bioinformatics sequence and genome analysis.

[B15] Nishikawa K (1983). Assessment of secondary-structure prediction of proteins comparison of computerized Chou-Fasman method with others. Biochim Biophys Acta.

[B16] Cuff JA, Barton GJ (1999). Evaluation and Improvement of Multiple Sequence Methods for Protein Secondary Structure Prediction. Proteins.

[B17] Minor DL, Kim PS (1994). Measurement of the beta-sheet forming propensities of amino acids. Nature.

[B18] Blaber M, Zhang XJ, Matthews BW (1993). Structural Basis of Amino Acid Alpha-Helix Propensity. Science.

[B19] Bystroff C, Garde S (2003). Helix propensities of short peptides: molecular dynamics versus bioinformatics. Proteins.

[B20] Jiang B, Guo T, Peng LW, Sun ZR (1998). Folding type-specific secondary structure propensities of amino acids, derived from α-Helical, β-Sheet, α/β, and α+β proteins of known structures. Biopolymers.

[B21] Barton GJ (1995). Protein secondary structure prediction. Curr Opin Struct Biol.

[B22] Viswanadhan VN, Denckla B, Weinstein JN (1991). New Joint Prediction Algorithm (Q7-JASEP) Improves the Prediction of Protein Secondary Structure. Biochemistry.

[B23] Levitt M, Chothia C (1976). Structural patterns in globular proteins. Nature.

[B24] Richardson JS (1981). The anatomy and taxonomy of protein structure. Adv Protein Chem.

[B25] Chou KC (1995). A novel approach to predicting protein structural classes in a (20-1)-D amino acid composition space. Proteins.

[B26] Chandonia JM, Karplus M (1995). Neural networks for secondary structure and structural class prediction. Protein Science.

[B27] Lio P (2003). Wavelets in bioinformatics and computational biology: state of art and perspectives. Bioinformatics.

[B28] Mandell AJ, Selz KA, Shlesinger MF (1997). Wavelet transformation of protein hydrophobicity sequences suggests their memberships in structural families. Physica A.

[B29] Dill KA (1990). Dominant forces in protein folding. Biochemistry.

[B30] Nozaki Y, Tanford C (1971). The solubility of amino acids and two glycine peptides in aqueous ethanol and dioxane solutions. Establishment of a hydrophobicity scale. J Biol Chem.

[B31] Eisenberg D, Weiss RM, Terwilliger TC (1984). The hydrophobic moment detects periodicity in protein hydrophobicity. Proc Natl Acad Sci.

[B32] Kabsch W, Sander C (1983). Dictionary of protein secondary structure: pattern recognition of hydrogen-bonded and geometrical features. Biopolymers.

[B33] Cuff JA, Barton GJ (1999). Evaluation and improvement of multiple sequence methods for protein secondary structure prediction. Proteins.

[B34] Rost B, Eyrich VA (2001). EVA: large-scale analysis of secondary structure prediction. Proteins.

[B35] Murzin AG, Brenner SE, Hubbard T, Chothia C (1995). SCOP: A structural classification of proteins database for the investigation of sequences and structures. J Mol Biol.

[B36] Rost B, Sander C (1993). Prediction of protein secondary structure at better than 70% accuracy. J Mol Biol.

[B37] Zemla A, Venclovas C, Fidelis K, Rost B (1999). A modified definition of SOV, a segment-based measure for protein secondary structure prediction assessment. Proteins.

